# 1037. Access to hospitalization for patients with severe COVID-19 during the first wave of the pandemic in Caracas, Venezuela.

**DOI:** 10.1093/ofid/ofac492.878

**Published:** 2022-12-15

**Authors:** Jose Orejas, David Forero, Daniela Mendoza, Oscar Omana, Fhabián S Carrión-Nessi, Maria Landaeta, Alejandro Diaz

**Affiliations:** Brigham and Women's Hospital, Brookline, Massachusetts; Hospital Universitario de Caracas, Caracas, Distrito Federal, Venezuela; Universidad Central de Venezuela, Caracas, Distrito Federal, Venezuela; Universidad Central de Venezuela, Caracas, Distrito Federal, Venezuela; Biomedical Research and Therapeutic Vaccines Institute, Ciudad Bolívar, Bolivar, Venezuela; Hospital Universitario de Caracas, Caracas, Distrito Federal, Venezuela; Brigham and Women's Hospital, Brookline, Massachusetts

## Abstract

**Background:**

The COVID-19 pandemic has overwhelmed health systems around the world, causing access problems. The patient referral system in Caracas, Venezuela has no interinstitutional coordination, patients are given a note by the referring center and thereafter seeking care relies on themselves. We studied primarily to what extent the Caracas healthcare system satisfied the demand for hospitalization of severe COVID-19 during periods of congestion, measured in proportion of patients not hospitalized; and secondly how difficult it was for patients with severe COVID-19 to meet their need of hospitalization, measured in number of days and visits made to health centers until hospitalization or desisting from further searching.

**Methods:**

This study was approved by national bioethics committee. We included all symptomatic patients who attended the University Hospital of Caracas COVID-19 testing center, whose nasopharyngeal swab PCR was positive for SARS-CoV-2, and whose SpO2 was 93% or less between 07/01/2020 and 07/24/2020, the period in which we referred patients due to unavailability of beds in our center.

**Results:**

Ninety-four patients were included, 66 (70%) were men, the mean age was 58 years (range, 25-87 years), the median number of comorbidities was 1 (range, 0-3), and the median SpO2 was 90% (range, 30%-93%). Fourty-three (46%) patients were never admitted following referral from our center. Referred patients required a median of 1 day (range, 1-15 days) and 2 visits (range, 1-41 visits) to being admitted, or a median of 2 days (range, 1-17 days) and 5 visits (range, 1-31 visits) to desisting from further searching for admission.

**Conclusion:**

These data show that the healthcare system did not meet the hospitalization needs of half of the patients with severe COVID-19 during the peak of the first wave of the pandemic in Caracas. The data also show that referred patients faced a major delay in being hospitalized, and that referred patients had to perform multiple visits to healthcare centers accross the city as a consequence of insufficient system capacity, increasing their urban mobility and therefore their probabilities of transmitting SARS-CoV-2 to others.

**Disclosures:**

**All Authors**: No reported disclosures.

